# Positive provocative testing and symptom evaluation for detecting upper extremity repetitive use injuries among endoscopists

**DOI:** 10.1055/a-2606-1076

**Published:** 2025-06-17

**Authors:** Gregory Toy, Elizabeth Cardell, John C Fang, Jamie Latham, Natalie Mudrow, Kathryn Byrne, Daryl Ramai

**Affiliations:** 1 14434 Gastroenterology, Hepatology, and Nutrition, University of Utah Health, Salt Lake City, United States; 2 14434 Department of Occupational & Recreational Therapies, University of Utah Health, Salt Lake City, United States; 3 14434 University of Utah Health, Salt Lake City, United States

**Keywords:** Quality and logistical aspects, Training, Performance and complications, Endoscopy Lower GI Tract, Endoscopy Upper GI Tract

## Abstract

**Background and study aims:**

Endoscopists have reported experiencing musculoskeletal pain, yet a comprehensive and objective investigation into repetitive use symptoms or injuries has not been conducted. We aimed to evaluate a cohort of endoscopists for upper extremity repetitive use injury.

**Methods:**

We employed a 43 author-developed questionnaire as well as the validated QuickDASH (Disability of Arm, Shoulder, Hand) questionnaire. Subjects were evaluated by occupational therapists to identify clinical evidence of injury. Demographic factors, reported symptoms, and signs of injury were then analyzed.

**Results:**

Overall, 34.3% reported experiencing pain while 17.1% reported numbness. In the prior week, 48.5% of participants had been bothered by pain, 11.4% felt tingling, 17.1% experienced interrupted sleep, and 17.1% reported limitations in work duties. Physical testing revealed that many endoscopists had below-normal strength in their right grip (48.6%) and left grip (42.9%), and 88.6% had below-normal pinch strengths for their age and gender. In addition, 71.4% of the group had at least one abnormal positive provocative test. Participants who reported numbness at night ( *P* = 0.015) and those who reported current pain ( *P* < 0.001) reported higher DASH disability scores. Current pain was also associated with performing 20+ procedures per week ( *P* = 0.007). Those with a positive provocative test of the neck or elbow were likely to have below-normal pinch ( *P* < 0.05) and grip strength ( *P* < 0.05). Performing ERCP 20% to 60% of the week was more likely to result in decreased bilateral pinch strength.

**Conclusions:**

Our study found a high prevalence of repetitive use injury symptoms among endoscopists, corroborated by objective physical examination and testing.

## Introduction

Despite changes in procedure volume, types, and endoscopist demographics, the design of the endoscope has remained relatively unchanged since the introduction of flexible video endoscopes in the 1980s 1 . Recent data indicate that the number of endoscopies performed by gastrointestinal endoscopists is on the rise 2 . Unfortunately, incidence of endoscopy-related injury (ERI) is also high, as reported by practitioners themselves. Some hypothesize that this to be due to lack of innovation in the ergonomics of endoscope design. A case control study comparing gastroenterology providers to non-procedure-oriented internal medicine specialists and subspecialists revealed that 74% of gastroenterology providers experienced musculoskeletal injury, which is more than twice the rate reported by the non-procedure-oriented providers at 35% 3 .

In an American Society for Gastrointestinal Endoscopy (ASGE) survey of gastroenterology providers, 78% of participants reported an ERI 4 . This was also the case with 48% of gastroenterology fellows, with 85% of those injuries occurring during their first year of training 5 . The survey authors suggest that the high prevalence of ERI may be linked to the number of procedures performed, because 82% of those who reported musculoskeletal injuries performed more than 20 procedures per week, whereas 74% reported performing endoscopy for more than 16 hours per week. Furthermore, a significantly higher proportion of endoscopists working in community practices reported higher ERI compared with those working in academic settings ( *P* = 0.006) 2 .

Although data on ERI and its associated risk factors offer valuable insights into the prevalence and potential causes of this condition, it is worth noting that the current data are solely based on surveys and self-reporting 5 6 7 . This approach has its limitations, including incomplete response rates and potential inaccuracies due to self-reporting bias. To date, no studies have utilized a validated disability survey combined with objective physical testing to assess a complete cohort of endoscopists for ERI. Our objective was to evaluate frequency of clinical symptoms of work-related injuries among endoscopists and analyze their impact on daily tasks, including those essential for endoscopy procedures. To gather comprehensive data on ERI, we utilized meticulous questionnaires, a validated disability survey, and physical assessments of bilateral upper extremity strength and provocative testing. Our study encompassed our entire academic gastroenterology division.

## Methods

Utilizing a psychometrically sound measure in conjunction with physical testing of each participant provides a more accurate, objective picture of individual participant status. The QuickDASH is a valid and reliable self-report instrument (11 items) used to assess perceived symptoms and effect on physical function in people with musculoskeletal injuries to the upper extremity 8 . Physical assessment involved provocative testing, which deliberately attempts to provoke or exacerbate symptoms to detect presence or absence of a musculoskeletal condition. This process is used in clinical practice to guide further assessments. For our purposes, presence or exacerbation of symptoms was an indication of a possible upper extremity musculoskeletal disorder.

### Survey and questionnaire

All faculty and fellows (n = 35) who perform endoscopy at a major university medical center completed a 43-question author-developed survey, which included background and demographic information such as gender, age, glove size, and volume, types and years performing procedures ( **Appendix Table 1** and **2** ). The survey questions were developed based on existing survey studies investigating ERI 2 5 6 7 , Participants also completed the validated QuickDASH (Disability of Arm, Shoulder, Hand) questionnaire, a Likert scale in which participants rate how well they perform several specific daily activities 8 ( **Appendix Table 3** ).

### Physical assessment

Study participants then underwent evaluation by an occupational therapist (OT) who assessed pain-free grip strength (right and left grip, tip to tip, tripod, and two-point pinches) with a Jamar hydraulic dynamometer (JLW Instruments, Chicago, Illinois, United States) and reported results in pounds (lb) and by comparison with age and gender-matched norms in tertiles (i.e. below normal, normal, and above normal). The OT also performed provocative testing on each subject’s back, shoulders, neck, elbows, wrists, and hands/thumbs ( Table 1 and **Appendix Table 4** ) 9 10 11 12 13 .

**Table:** **Table 1** Summary of provocative tests.*

Neck	Cervical screen, Spurling test
Shoulder	Posture screen, rounded shoulders
Back	Posture screen, pelvic tilt
Elbow	Resistive extension, pain-free grip
Wrist	Finklestein, Phalen’s, carpal compression
Hand/thumb	CMC Grind Test
*For a more detailed description, see **Appendix Table 1** .	

### Statistical analysis

For purposes of data analysis, a chi squared or Fisher’s exact test was used for categorical variables and Wilcoxson rank sum test was used for continuous variables. Continuous variables of years performing procedure, time spent scoping per week, number of procedures per week, and percent of time spent doing esophagogastroduodenoscopy (EGD), colonoscopy, endoscopic retrograde cholangiopancreatography (ERCP), and endoscopic ultrasound (EUS) were categorized. Differences in strength testing between genders were analyzed. Presence and absence of the binary outcomes numbness/tingling at night, experiencing current pain, and modification of endoscopy technique was analyzed against other risk factors from the surveys and physical testing. In addition, presence of a positive provocative test was analyzed against the other risk factors. Finally, presence of each positive provocative test (neck, shoulder, back, elbow, wrist, hand/thumb) was analyzed against the other risk factors.

To identify risk factors that influence continuous outcomes provided by strength testing, lasso regression models were built. The models used a leave-one-out cross validation to choose the best tuning parameter that yielded the least mean squared error or the least mean squared error within one standard error for models that selected large risk factors but were not significant. At the best tuning parameter, the lasso model was fitted to select which risk factor profiles influence the outcome. A linear regression model was fitted to obtain the unbiased effect and significance of these identified risk factors to continuous outcomes. In addition, multicollinearity was assessed with the variance inflation factor. Significance was defined as *P* < 0.05.

Odds ratios were reported for the logistic model with 95% confidence intervals and *P* values, and mean estimates with associated 95% confidence intervals and *P* values were given for the continuous outcomes. All statistical analysis was run using R v4.1.1 (R Foundation for Statistical Computing, Vienna, Austria), specifically using the glm, lm, and glmnet functions. This study was approved by the Institutional Review Board of the University of Utah.

## Results

### Demographics

Overall, the sample consisted of 26 males (74%) and nine females (26%) with 26 faculty members (74%) and nine fellows (26%). The average (standard deviation [SD]) age was 44.6 (10.8) and body mass index (SD) was 24.5 (3.0) ( Table 2 ).

**Table:** **Table 2** Demographics data for study participants (N = 35).

**Variable**	**Summary (N = 35)**
**Gender**	
Female	9 (25.7%)
Male	26 (74.3%)
**Age**	
Mean (SD)	44.6 (10.8)
Median (IQR)	42.0 (35.5–50.5)
Range	(30.0–71.0)
**Glove size**	
Medium	15 (42.9%)
Small	8 (22.9%)
Large/Extra large	12 (34.3%)
**Dominant hand**	
Left	1 (2.9%)
Right	34 (97.1%)
**Years performing procedures**	
0–5	10 (28.6%)
6–10	11 (31.4%)
11–15	2 (5.7%)
16–20	3 (8.6%)
21–25	4 (11.4%)
26–30	3 (8.6%)
30–35	2 (5.7%)
**BMI**	
Mean (SD)	24.5 (3.0)
Median (IQR)	24.4 (22.2–25.8)
Range	(17.7–30.7)
**Time spent scoping**	
< 10 hours	4 (11.4%)
10–20 hours	12 (34.3%)
21–30 hours	14 (40%)
30+ hours	5 (14.3%)
**# of procedures per week**	
0–20	11 (31.4%)
20–40	19 (54.3%)
40–60	4 (11.4%)
80+	1 (2.9%)
**Type of procedure**	
Colonoscopy	31 (88.6%)
EGD/EUS	1 (2.9%)
ERCP	3 (8.6%)
**Colonoscopy time**	
0%-40%	9 (25.7%)
40%-60%	12 (34.3%)
60%-100%	14 (40%)
**ERCP time**	
0%-20%	32 (91.4%)
20%-60%	3 (8.6%)
**EGD time**	
0%-20%	6 (17.1%)
20%-40%	17 (48.6%)
40%-60%	7 (20%)
60%-100%	5 (14.3%)
**EUS time**	
0%-20%	33 (94.3%)
20%-40%	2 (5.7%)
**Physical activity level**	
Moderate	20 (57.1%)
Mild/none	5 (14.3%)
Extreme	10 (28.6%)
BMI, body mass index; EGD, esophagogastroduodenoscopy; ERCP, Endoscopic retrograde cholangiopancreatography; EUS, endoscopic ultrasound; IQR, interquartile range; SD, standard deviation.	

### Survey responses

The response rate to our survey was 100% because each of the 35 members of the University of Utah Gastroenterology Department at the time responded to the survey. Most of the providers reported performing procedures for 6 to 10 years (31.4%), 20 to 40 procedures per week (54.3%), and 21 to 30 hours (40%) per week. The most common glove size was medium as reported by 42.9% of participants. Only seven participants (20%) reported that they received ergonomics training during fellowship. Thirty-four participants (97%) reported making environmental modifications prior to starting a procedure such as changing bed position, screen position, or scope tower position. A majority of the participants reported making personal modifications with 11 (31.4%) performing stretching or strength exercises, one (2.9%) taking more or longer breaks, and four (11.4%) performing fewer procedures. Eight participants (22.9%) reported making “other changes” which were non-endoscopic-related including yoga, core training, and physical therapy. Seventeen (48.6%) reported making no personal modifications. In terms of symptoms, over one-third (34.3%) of the participants reported current pain, six participants (17.1%) reported numbness/tingling at night (3 in an ulnar nerve distribution, 1 in a medial nerve distribution, 1 at the righ metacarpophalangeal joint, and 1 from a broken clavicle) and three participants (8.8%) reported numbness/tingling while performing procedures (all reported shooting pain down the arm into the wrist and hand) ( Table 3 ). Of those who reported pain related to endoscopy (n = 12) only two (16%) had received ergonomic training in the past, whereas five (41.7%) reported the symptoms for 0 to 5 years.

**Table:** **Table 3** Survey question responses (N = 35).

Presence of numbness or tingling at night	6 (17.1%)
Presence of numbness or tingling while performing a procedure	3 (8.8%)
Any current pain	12 (34.3%)
Modified endoscopy technique	6 (17.1%)
Modified body position	32 (91.4%)
Years with symptoms (N = 12)	
0–5	5 (41.7%)
6–10	2 (16.7%)
11–15	3 (25%)
16–20	1 (8.3%)
21–25	0 (0%)
26–30	1 (8.3%)
30+	0 (0%)

### QuickDASH disability scores

Mean and median QuickDASH scores were 6.0 (SD 8.9) and 2.3 (interquartile range 1.0–3.0), respectively. These scores are lower (show less disability) than normative scores of a similar age group to our sample 14 . Using QuickDASH, participants identified functional limitations experienced in the week prior due to ERI. Of the participants, 48.5% had been bothered by pain, 11.4% felt tingling, 17.1% had interrupted sleep, and 17.1% experienced limitations in work duties. Participants also rated the amount of difficulty (1 = no difficulty to 5 = unable) they had with specific daily tasks that require common upper extremity movements. Results indicate 24 participants rated at least mild difficulty with one of the tasks, the most common being opening a jar (28.6%), recreation involving impact (20%), heavy household chores (20%), and washing their back (20%) ( Table 4 ).

**Table:** **Table 4** Results of Quick DASH survey.

DASH question	# Participants reporting at least mild difficulty
Ability to open a tight/new jar in the past week	10
Heavy household chores in the past week	7
Carry a shopping bag or briefcase in the last week	3
Wash your back in the past week	7
Recreational activities which take some force or impact through arm, shoulder and back in the past week	10
During the past week to what extent has your arm, shoulder, or hand problem interfered with your social activities with friends, family, neighbors or groups?	4
During the past week, were you limited in your work or other regular daily activities as a result of your arm, shoulder or hand problem?	6
Severity of arm, shoulder, or hand pain in the last week	17
Severity of tingling (pins and needles) in your arm, shoulder, hand	4
During the past week, how much difficulty have you had sleeping because of the pain in your arm, shoulder or hand?	6
Total number of participants that rated mild on at least one activity	24
DASH, Disability of Arm, Shoulder, Hand.	

### Provocation and strength testing

Results of upper extremity provocative testing showed 71.4% had at least one positive provocative test, with the highest percentage of positive tests in shoulder and back ( Table 4 ). Compared with norms for both age and gender, many endoscopists recorded below normal pain-free strength; 48.8% had reduced right grip, 42.9% had reduced left grip and the majority (45%-80%) had a decrease in at least one of the pinch strength tests ( Table 5 ). When comparing pain-free strength between males and females in the sample, males had significantly higher strength for both right and left hands for all tests conducted (right and left grip, tip to tip, tripod, and two-point pinches) ( Table 6 ).

**Table:** **Table 5** Results of provocative testing.

Any positive provocative test	Yes	25 (71.4%)
	No	10 (28.6%)
Neck provocative test	Negative	31 (88.6%)
	Positive	4 (11.4%)
Shoulder provocative test	Negative	16 (45.7%)
	Positive	19 (54.3%)
Back provocative test	Negative	23 (65.7%)
	Positive	12 (34.3%)
Elbow provocative test	Negative	29 (82.9%)
	Positive	6 (17.1%)
Wrist provocative test	Negative	22 (62.9%)
	Positive	13 (37.1%)
Hand/thumb provocative test	Negative	32 (91.4%)
Any positive provocative test	Positive	3 (8.6%)

**Table:** **Table 6** Grip strength testing by gender.

**Variable**	**Overall (N = 35)**	**Male (N = 26)**	**Female (N = 9)**	***P* value **
Right (R) grip avg (lb) Mean (SD)	82.3 (23.6)	89.8 (21.1)	60.7 (16.8)	-
Median (IQR)	85.3 (66.7, 98.0)	88.0 (80.3, 103.6)	62.0 (44.0, 72.7)	< 0.001*
Range	(38.3–126.7)	(49.3–126.7)	(38.3–85.7)	-
Left (L) grip avg (lb) mean (SD)	77.2 (21.3)	84.2 (17.2)	57.0 (19.4)	-
Median (IQR)	81.7 (61.5–94.5)	85.2 (74.5- 97.4)	52.0 (43.3- 68.0)	< 0.001*
Range	(32.0–114.7)	(44.7–114.7)	(32.0–89.0)	-
L-lat pinch mean (SD)	18.6 (4.8)	20.4 (3.7)	13.3 (3.7)	-
Median (IQR)	18.0 (16.0–21.5)	20.0 (18.0–22.8)	14.0 (10.0- 17.0)	< 0.001*
Range	(9.0–27.0)	(15.0–27.0)	(9.0–18.0)	-
R-lat pinch mean (SD)	19.2 (4.7)	20.8 (3.7)	14.6 (3.9)	-
Median (IQR)	20.0 (16.0–22.0)	20.0 (18.2–22.8)	14.0 (12.0–17.0)	< 0.001*
Range	(9.0–30.0)	(15.0–30.0)	(9.0–20.0)	-
L-tripod mean (SD)	13.9 (3.8)	14.8 (3.6)	11.2 (3.3)	-
Median (IQR)	14.0 (12.0–16.5)	14.5 (12.0–17.0)	12.0 (10.0–14.0)	0.026*
Range	(5.0–20.0)	(8.0–20.0)	(5.0–15.0)	-
R-tripod mean (SD)	14.7 (3.7)	15.6 (3.6)	12.0 (3.0)	-
Median (IQR)	15.0 (12.5–16.5)	15.5 (13.2–18.0)	13.0 (10.0–15.0)	0.015*
Range	(8.0–23.0)	(10.0–23.0)	(8.0–15.0)	-
L-2 point mean (SD)	10.2 (3.2)	11.0 (3.1)	7.8 (1.9)	-
Median (IQR)	10.0 (8.5–11.5)	10.0 (9.0–13.0)	9.0 (6.0–9.0)	0.003*
Range	(5.0–18.0)	(6.0–18.0)	(5.0–10.0)	-
R-2 point mean (SD)	11.1 (3.1)	11.9 (3.0)	8.8 (2.0)	-
Median (IQR)	10.0 (9.0–13.0)	12.0 (9.2–14.0)	10.0 (8.0–10.0)	0.012*
Range	(5.0–18.0)	(8.0–18.0)	(5.0–11.0)	-
*Exact Wilcoxon rank sum test. IQR, interquartile range; SD, standard deviation.				

### Symptoms and risk factors

Following analysis associating symptoms and risk factors, the results worth noting are related to numbness/tingling at night, current pain, and modifying endoscopic techniques ( **Appendix Tables 5, 6, 7,** and **8** ). Participants who reported numbness/tingling at night were significantly more likely to have current pain, have modified their endoscopic technique, perform a higher number of procedures during per week, and have positive elbow and wrist provocative tests with lower two-point and left grip strengths. They also had significantly higher average QuickDASH disability scores (13.5 vs. 4.4, *P* = 0.015).

Participants reporting current pain were significantly more likely to have numbness/tingling at night (50% vs. 0%, *P* < 0.001), modify their endoscopic technique (50% vs. 0%, *P* < 0.001), and perform higher number of procedures during the week ( *P* = 0.007) and had a higher average QuickDASH disability score (10.0 vs. 3.9, *P* = 0.042). Conversely, those participants who did not report current pain were significantly more likely to have normal or better grip strength ( *P* = 0.032).

Participants who noted modifying their endoscopic technique were significantly more likely to report numbness/tingling at night, current pain, were also more likely to perform more procedures per week ( *P* = 0.009), and to have fewer years of experience ( *P* = 0.004).

### Positive provocative test correlation

Participants with a positive neck provocative test were more likely to have decreased scores on right lateral pinch ( *P* < 0.001), right ( *P* = 0.028) and left ( *P* = 0.018) tripod, and right two-point grip strengths ( *P* = 0.015) with below normal right grip strength ( *P* = 0.034). A positive shoulder provocative test was associated with a positive back provocative test ( *P* = 0.013) and decreased time per week spent performing endoscopy ( *P* = 0.045). A positive back provocative test was associated with positive shoulder ( *P* = 0.013) and hand/thumb ( *P* = 0.034) provocative tests as well as performing fewer procedures per week ( *P* = 0.042). A positive elbow provocative test was associated with having numbness/tingling at night (50% vs 10.3%, *P* = 0.049) and positive neck provocative test ( *P* = 0.011). In addition, they had decreased scores on bilateral lateral pinch, bilateral two-point and left tripod grip strength and were more likely to have below normal bilateral grip strength. A positive wrist provocative test was associated with below normal left grip scores ( *P* = 0.029). Positive hand/thumb provocative tests were associated with a positive back provocative test ( *P* = 0.034).

### Lasso regression

Lasso variable selection was performed on all the strength tests ( **Appendix Tables 9, 10, 11, 12, 13, 14, 15, 16,** and **17** ). Comparisons of subgroups in our sample were made against other subgroups in our sample. Increased age was associated with significantly lower tripod scores on both the right ( *P* < 0.001) and left side ( *P* = 0.01) and significantly lower two-point test scores for both the right ( *P* = 0.03) and left ( *P* = 0.01). There was significantly decreased right lateral pinch for those performing fewer than 20 procedures per week ( *P* = 0.01). Providers who spent less than 10 hours scoping per week had decreased two-point pinch scores with the right-sided scores reaching significance ( *P* = 0.01). These providers also had lower tripod scores. This reached significance for the right tripod score ( *P* < 0.001). Providers with a large or extra-large glove size had higher two-point pinch scores on the left side ( *P* = 0.03).

For the lasso variable selection of QuickDASH disability score, there were no input variables that reached significance. However, there was a trend toward significance because endoscopists with 0 to 5 years performing procedures were found to have lower disability scores ( *P* = 0.12) whereas those in the middle years performing procedures (16–20 years) and number of procedures weekly (40–60 procedures) had higher disability scores ( *P* = 0.08 and *P* = 0.07, respectively).

For the lasso variable selection of average pain-free grip strength on the right and left, there again were no input variables that reached significance but there was a trend that endoscopists who spent 26 to 30 years performing procedures had a higher average pain-free grip strength ( *P* = 0.1 for right grip strength and *P* = 0.09 for left grip strength).

## Discussion

Although several survey-based studies have attempted to quantify the prevalence and risk factors of ERI in endoscopists, to our knowledge, there have not been any studies that used a validated disability survey or objective measures to investigate ERI symptoms. This study is the first to correlate subjective findings from survey data with objective data from provocative testing and examination. There are several unique findings from this study.

The literature reports that some of the most commonly reported ERIs present as carpal tunnel syndrome, de Quervain’s tenosynovitis, and lateral epicondylitis 6 . Our study supports this by showing a significant association between endoscopists who reported numbness/tingling and having a positive wrist or elbow provocative test, indicating possible presence of one of these ERIs. In addition, of the endoscopists who reported modifying their endoscopy techniques, significantly more reported numbness/tingling at night or pain. Other positive associations related to this are high procedure volume and less experience. This may suggest that endoscopists with less experience who are having symptoms are more open to modifying their endoscopic techniques in an attempt to alleviate their pain because their endoscopic techniques are less ingrained than someone who has been practicing for decades. The relationship we noted between grip strength and positive neck and elbow provocative tests is likely related to the upper extremity kinetic chain, which often causes referred neck pain to be experienced in the elbow, leading to decreased grip strength 15 16 . However, it is also plausible that endoscopists with weak grip strength could be more likely to sustain these injuries because they compensate with neck and elbow movements.

Analysis of our pain-free grip strength data found that about half of participants had grip strength at least one SD below average and about three-quarters of participants had grip strength below average for their age and/or gender. Because participants were asked to stop the grip and pinch tests upon feeling pain and comparative norms are set using maximum effort, caution should be used when interpreting our outcomes. Our results indicate that the majority of our participants experience pain at a grip force less than they would be able to produce if pain were not a factor. This suggests that the majority have pain with force-related tasks, similar to those needed during an endoscopy procedure. Another possible interpretation could be that weakened grip strength is exacerbated by the design of the endoscope. In one study of gastroenterology fellows, most participants with a glove size < 6.5 felt that the endoscope was too large and impeded their ability to effectively learn endoscopy 17 . Endoscopists who have hands that are too small for the endoscope are more likely to use a suboptimal grip, which decreases their ability to produce voluntary force 11 .

The data gathered in this study reinforce some previously noted associations regarding ERI. First, there was a significant positive association between the number of procedures performed per week and reports of pain. Ridtitid et al. found a similar association between procedure volume and ERI 2 . Our study notably did not find an association between years performing endoscopy and numbness/tingling at night or current pain, although this may have been due to our smaller number of participants. Although women had significantly lower pain-free grip strength than men, there was no association between gender and numbness/tingling, pain, or having a positive provocative test. However, as an absolute percentage, women did report more ERI symptoms. Recent studies have shown conflicting results regarding sex differences in ERI. Two studies found that there was no difference in prevalence of ERI between men and women but noted that women reported more neck and shoulder pain after procedures 5 18 . However, other recent studies have found an increased prevalence of ERI in women 19 20 21 .

Some unexpected findings included the association between a positive shoulder provocative test and decreased time per week performing endoscopy and a positive back provocative test and performing fewer procedures per week. In addition, the most experienced endoscopists (26–30 years performing procedures) had higher average pain-free grip strength. These findings would seem to go against findings in the literature suggesting that performing more procedures leads to higher ERI. In this descriptive study, we can only theorize that providers who spend more time performing procedures or have been performing procedures for a long time may have built up muscle strength that overcomes the initial detriment of performing a high volume of endoscopy either in a snapshot in time or over years. This would be an interesting hypothesis to be studied in future studies.

According to the literature, interventions aimed at preventing work-related musculoskeletal disorders (WMSD) have had varied success. These interventions commonly include ergonomic training, occasionally combined with exercise. A related Cochrane Review determined that although ergonomic training did not decrease pain in the short term, training was successful in the long term 22 . A randomized clinical trial investigating the effectiveness of a WMSD prevention program for surgeons reported that the combination of ergonomic training and physical exercise was successful in reducing back pain (66.2% vs 50.0%, *P* = 0.04) after 6 months 23 .

In our study, of those who reported incorporating strengthening or stretching (n = 11) into their procedure modifications, only four reported pain in the last week, only one of whom reported pain during procedures. This suggests that strengthening/stretching may be a useful modification during procedures. In a study related to WMSD in gastroenterology fellows, the authors reported that few fellows received training but those who did were less likely to report presence of WMSD 6 . This suggests that the training was effective. Yet in our study, of the seven participants who received ergonomic training in the past, five (71.4%) still had a positive provocative test, suggesting that the training was ineffective.

Some limitations of our study stem from the applicability of our sample to other practice environments. Although our sample included endoscopists who work in a variety of academic settings, it may not reflect gastroenterologists in private practice settings, typically with higher procedure volumes and more uniform procedure types. Our study addressed the non-response bias through surveying and examining every member of a gastroenterology division at a large academic medical center, but although that still achieved only a moderate-sized sample of 35, it may not have represented all demographic subgroups with other possible contributing factors.

In addition, the provocative tests that were administered are those typically used to screen for and not diagnose repetitive use injuries. Due to small sample size, we were unable to complete some analysis that might be interesting, such as comparing right dominant to left, male vs female. Furthermore, additional questions arose which we could not answer due to the scope of this paper. Do subjects have decreased strength because of pain and numbness, or do they have pain and numbness because of decreased strength?

However, our study has notable strengths, including the spectrum of data captured. This study used data from both a questionnaire based on previous survey studies and also added a validated disability survey, which had not been performed in previous studies. In addition, our study utilized occupational therapists to collect objective, quantifiable data using provocative tests, physical examination, and strength testing. To this end, this is the first study of ERI that correlated objective findings with reported survey data. Our sample also contains endoscopists from the full spectrum of experience in terms of years performing procedures and type of procedures from standard upper and lower endoscopy to therapeutic procedures.

There are several opportunities for future studies relating to ERI. This can include introducing an intervention where participants would get a baseline evaluation with the surveys and provocative tests used and a reevaluation after the intervention. A control group of faculty who do not scope could be introduced to compare the responses and provocative tests results of practitioners who do not practice endoscopy as a baseline. Additional provocative tests involving hips, knees, ankles, and feet could be added because over the last few years, we have started to recognize lower extremity injuries as a part of ERI.

## Conclusions

The data in our study show a high prevalence of reported ERI symptoms when an entire cohort of endoscopists were assessed. In addition, providers with symptoms had significantly higher disability scores. Using objective testing, provocative exams performed by occupational therapists showed a high incidence of physical symptoms and decreased strength. Further studies should expand this testing to larger groups of endoscopists in addition to other practice settings, with the ultimate goal of developing awareness about and interventions to prevent and manage ERI.

## Supplementary Material

Supplementary Material Zusatzmaterial
