# Supplementary material for: Positive provocative testing and symptom evaluation for detecting upper extremity repetitive use injuries among endoscopists
Source: Endosc Int Open. 2025 Jun 17;13:a26061076. doi: 10.1055/a-2606-1076 (PMC12223948; doi:10.1055/a-2606-1076)
Supplement: Supplementary file 1 — Supplementary Material [file 10-1055-a-2606-1076_26103511.pdf]

Appendix Table 1 Author-developed survey.

**Demographics**
Gender
Age
Height
Weight
How physically active would you say you are?
Are you left-handed or right-handed?
**Percent of practice performing the following procedures**
EGD
Colonoscopy
ERCP
EUS
Other endoscopic procedures
**Endoscopy specific questions**
How long have you been performing endoscopic procedures? (in years)
On average, how many hours per week do you spend performing endoscopic procedures?
On average, how many endoscopic procedures do you perform each week?
Did you receive ergonomics training related to endoscopy during your fellowship?
What factors do you evaluate/modify prior to starting a procedure? (Select all that apply)
Do you feel that the endoscopes' size, design, and function are well-suited for your body/hand type?
What size glove do you wear?
What modifications (if any) have you made to your endoscopy technique to prevent pain or injury?
Do you currently or have you ever had any chronic pain or injuries you feel are affiliated with performing endoscopic procedures? (Yes or No)

EGD, esophagogastroduodenoscopy; ERCP, endoscopic retrograde cholangiopancreatography; EUS, endoscopic ultrasound.

Appendix Table 2 Secondary survey questions if participant endorsed pain.

Is your chronic pain or injury currently causing symptoms?
Have you had any previous upper extremity musculoskeletal trauma from something other than endoscopy?
If yes, what kind of musculoskeletal injury?
Have you had a spine injury?
If yes, what kind of spine injury?
What is the highest rated pain you experience on a procedure day?
0 = No pain
10 = Worst pain ever
**Questions about the location and symptoms of your pain or injury**
Descriptive nature of pain?
location of pain/injury continued (check all that apply)
which of the following do you attribute to most likely being the cause of your pain/injury? (select all that apply)
Do you feel that your pain/injury is or was caused by repetitively performing endoscopic procedures?
When is your pain/injury most evident?
How much did/does your pain/injury affect you?
How long have you had these symptoms? (in years)
After how many years (in relation to the start of your scoping experience) did the pain/injury become present?
Have you ever taken any time off of work because of your pain/injury?
Have you seen a medical professional about assessment or treatment of this pain or injury?
What treatment(s) have you received for your pain/injury? (Select all that apply)
Have you modified your endoscopy technique at all due to your pain/injury?
Have you had any previous injuries to the affected body part outside of endoscopy? (i.e. due to physical activity or trauma)

Appendix Table 3 QuickDASH questionnaire.

	No difficulty	Mild difficulty	Moderate difficulty	Severe difficulty	Unable
Open a tight or new jar					
Do heavy household chores					
Carry a shopping bag or briefcase					
Wash your back					
Use a knife to cut food					
Recreational activities in which you take some force or impact through your arm, shoulder or hand					
	**Not at all**	**Slightly**	**Moderately**	**Quite a bit**	**Extremely**
During the past week, to what extent has your arm, shoulder or hand problem interfered with your normal social activities with family, friends, neighbours or groups?					
	**Not limited at all**	**Slightly limited**	**Moderately limited**	**Very limited**	**Unable**
During the past week, were you limited in your work or other regular daily activities as a result of your arm, shoulder or hand problem?					
	**None**	**Mild**	**Moderate**	**Severe**	**Extreme**
Arm, shoulder or hand pain.					
Tingling (pins and needles) in your arm, shoulder or hand.					
	**No difficulty**	**Mild difficulty**	**Moderate difficulty**	**Severe difficulty**	**So much difficulty that I**

	**can't sleep**
During the past week, how much difficulty have you had sleeping because of the pain in your arm, shoulder or hand?	

DASH, Disability of Arm, Shoulder, Hand.

Appendix Table 4 Description of provocative tests.

Test	Description
Cervical screen	Examine active range of motion in all directions. Positive test: limitation in range of motion
Spurling test	Neck in extension and lateral flexion; apply axial compression. Performed to right and left. Positive test: presence of pain or paresthesia radiating down arm
Posture screen	Examine posture during standing. Positive test: presence of anterior/posterior pelvic tilt or kyphosis
Rounded shoulders	Examine shoulders in standing. Positive test: rounding posture/forward head
Resistive extension	Resistance applied to wrist extension with shoulder in 90 degrees flexion, elbow extended, and fingers flexed. Performed bilaterally. Positive test: Presence of pain with resistance
Pain-free grip	Measure grip strength with handheld Jamar hydraulic dynamometer, end point when pain is felt. Performed bilaterally.
Finklestein test	Supported forearm in neutral, wrist in ulnar deviation. Apply pressure to ulnar deviation; passive thumb flexion. Performed bilaterally. Positive test: presence of pain over styloid process with either movement
Phalen's test	flexed elbows, maximal flexion of wrist with dorsal surfaces pushing together at chest level. Hold this position for 60 seconds. Positive test: tingling in first three digits or increase of symptoms
Carpal compression	forearm is supinated; examiner applies direct pressure over the carpal tunnel for 1 minute. Performed bilaterally. Positive test: pain, paresthesia, numbness distal to compression
CMC grind test	grip the patient's thumb metacarpal and move it in a circle; loading it with gentle axial forces. Performed bilaterally. Positive test: pain or discomfort

CMC, carpometacarpal.

Appendix Table 5 Association between risk factors and presence of numbness or tingling at night.

Variable	Yes (N = 6)	No (N = 29)	*P* value
**Gender:** Female	2 (33.3%)	7 (24.1%)	0.64*
Male	4 (66.7%)	22 (75.9%)	-
**Age:** Mean (SD)	51.2 (13.4)	43.2 (10.0)	0.22†
Median (IQR)	50.0 (42.2, 58.5)	41.0 (35.0, 46.0)	-
Range	(35.0, 71.0)	(30.0, 64.0)	-
**Glove size:** Medium	2 (33.3%)	13 (44.8%)	0.73*
Small	1 (16.7%)	7 (24.1%)	-
Large/extra large	3 (50%)	9 (31%)	-
**Dominant hand:** Left	1 (16.7%)	0 (0%)	0.17*
Right	5 (83.3%)	29 (100%)	-
**Years performing procedures:** 0-5	1 (16.7%)	9 (31%)	0.68*
6-10	2 (33.3%)	9 (31%)	-
11-15	0 (0%)	2 (6.9%)	-
16-20	0 (0%)	3 (10.3%)	-
21-25	1 (16.7%)	3 (10.3%)	-
26-30	1 (16.7%)	2 (6.9%)	-
30-35	1 (16.7%)	1 (3.4%)	-
**BMI:** Mean (SD)	24.4 (3.5)	24.5 (3.0)	0.91†
Median (IQR)	23.9 (22.0, 24.9)	24.4 (22.4, 25.8)	-
Range	(21.0, 30.7)	(17.7, 29.5)	-
**Time spent scoping:** < 10 hours	0 (0%)	4 (13.8%)	0.85*
10-20 hours	3 (50%)	9 (31%)	-
21-30 hours	2 (33.3%)	12 (41.4%)	-
30+ hours	1 (16.7%)	4 (13.8%)	-
**Number procedures per week:** 0-20	1 (16.7%)	10 (34.5%)	0.39*
20-40	3 (50%)	16 (55.2%)	-
40-60	2 (33.3%)	2 (6.9%)	-
80+	0 (0%)	1 (3.4%)	-
**Type of procedure:** Colonoscopy	5 (83.3%)	26 (89.7%)	0.55*
EGD/EUS	0 (0%)	1 (3.4%)	-
ERCP	1 (16.7%)	2 (6.9%)	-
**Colonoscopy time:** 0%-40%	2 (33.3%)	7 (24.1%)	1.00*
40%-60%	2 (33.3%)	10 (34.5%)	-
60%-100%	2 (33.3%)	12 (41.4%)	-

Variable	Yes (N = 6)	No (N = 29)	*P* value
**ERCP time:** 0%-20%	5 (83.3%)	27 (93.1%)	0.44*
20%-60%	1 (16.7%)	2 (6.9%)	-
**EGD time:** 0%-20%	2 (33.3%)	4 (13.8%)	0.36*
20%-40%	2 (33.3%)	15 (51.7%)	-
40%-60%	2 (33.3%)	5 (17.2%)	-
60%-100%	0 (0%)	5 (17.2%)	-
**EUS time:** 0%-20%	6 (100%)	27 (93.1%)	1.00*
20%-40%	0 (0%)	2 (6.9%)	-
**Modifications - body position:** No	1 (16.7%)	2 (6.9%)	0.44*
Yes	5 (83.3%)	27 (93.1%)	-
**Activity level:** Moderate	4 (66.7%)	16 (55.2%)	0.69*
Mild/none	0 (0%)	5 (17.2%)	-
Extreme	2 (33.3%)	8 (27.6%)	-
**At what year in:** 0-5	3 (50%)	2 (33.3%)	0.61*
6-10	0 (0%)	2 (33.3%)	-
11-15	2 (33.3%)	1 (16.7%)	-
16-20	1 (16.7%)	0 (0%)	-
26-30	0 (0%)	1 (16.7%)	-
**QD disability Sx score:** Mean (SD)	13.6 (6.4)	4.4 (8.6)	0.015†
Median (IQR)	12.5 (9.7, 15.3)	2.3 (0.0, 2.3)	-
Range	(6.8, 25.0)	(0.0, 43.2)	-
**presence of numbness or tingling while performing a procedure:** No	4 (80%)	27 (93.1%)	0.39*
Yes	1 (20%)	2 (6.9%)	-
**Any current pain:** No	0 (0%)	23 (79.3%)	< 0.001*
Yes	6 (100%)	6 (20.7%)	-
**Modified:** No	2 (33.3%)	27 (93.1%)	0.004*
Yes	4 (66.7%)	2 (6.9%)	-
**Any positive provocative test:** No	1 (16.7%)	9 (31%)	0.65*
Yes	5 (83.3%)	20 (69%)	-
**Neck provocative test:** Negative	4 (66.7%)	27 (93.1%)	0.13*
Positive	2 (33.3%)	2 (6.9%)	-
**Shoulder provocative test:** Negative	2 (33.3%)	14 (48.3%)	0.67*
Positive	4 (66.7%)	15 (51.7%)	-

Variable	Yes (N = 6)	No (N = 29)	*P* value
**Back provocative test:** Negative	4 (66.7%)	19 (65.5%)	1.00*
Positive	2 (33.3%)	10 (34.5%)	-
**Elbow provocative test:** Negative	3 (50%)	26 (89.7%)	0.049*
Positive	3 (50%)	3 (10.3%)	-
**Wrist provocative test:** Negative	1 (16.7%)	21 (72.4%)	0.019*
Positive	5 (83.3%)	8 (27.6%)	-
**Hand/thumb provocative test:** Negative	4 (66.7%)	28 (96.6%)	0.07*
Positive	2 (33.3%)	1 (3.4%)	-
**R-grip avg (lb):** Mean (SD)	85.1 (22.9)	81.8 (24.1)	0.76†
Median (IQR)	85.5 (70.1-86.2)	83.3 (68.3-102.3)	-
Range	(62.0- 126.0)	(38.3- 126.7)	-
**L-grip avg (lb):** Mean (SD)	82.9 (17.1)	76.0 (22.1)	0.42†
Median (IQR)	87.2 (80.1-92.2)	79.0 (60.0-95.7)	-
Range	(51.3- 100.0)	(32.0- 114.7)	-
**L-Lat pinch:** Mean (SD)	17.8 (2.6)	18.7 (5.2)	0.55†
Median (IQR)	17.5 (17.0-18.8)	18.0 (16.0-22.0)	-
Range	(14.0- 22.0)	(9.0- 27.0)	-
**R-lat pinch:** Mean (SD)	18.5 (3.8)	19.4 (4.9)	0.64†
Median (IQR)	18.5 (15.5-20.8)	20.0 (16.0-22.0)	-
Range	(14.0- 24.0)	(9.0- 30.0)	-
**L-tripod:** Mean (SD)	13.0 (3.0)	14.0 (4.0)	0.48†
Median (IQR)	13.5 (12.2-14.0)	14.0 (12.0-17.0)	-
Range	(8.0- 17.0)	(5.0- 20.0)	-
**R-tripod:** Mean (SD)	14.8 (2.8)	14.7 (3.9)	0.90†
Median (IQR)	14.5 (13.2-15.0)	15.0 (12.0-17.0)	-
Range	(12.0- 20.0)	(8.0- 23.0)	-
**L-2 point:** Mean (SD)	9.5 (1.9)	10.3 (3.4)	0.41†
Median (IQR)	9.0 (8.2- 9.8)	10.0 (9.0- 12.0)	-
Range	(8.0- 13.0)	(5.0- 18.0)	-
**R-2 point:** Mean (SD)	9.3 (1.5)	11.4 (3.2)	0.025†

Variable	Yes (N = 6)	No (N = 29)	*P* value
Median (IQR)	9.0 (8.2- 9.8)	11.0 (10.0- 14.0)	-
Range	(8.0- 12.0)	(5.0- 18.0)	-
**R-grip cat:** Below Normal	3 (50%)	5 (17.2%)	0.31*
Normal	2 (33.3%)	15 (51.7%)	-
Above normal	1 (16.7%)	9 (31%)	-
**L-grip cat:** Below normal	3 (50%)	7 (24.1%)	0.032*
Normal	0 (0%)	15 (51.7%)	-
Above normal	3 (50%)	7 (24.1%)	-
**L-lat pinch cat:** Below normal	4 (66.7%)	22 (75.9%)	0.64*
Normal	2 (33.3%)	7 (24.1%)	-
**R-lat pinch cat:** Below normal	5 (83.3%)	23 (79.3%)	1.00*
Normal	1 (16.7%)	6 (20.7%)	-
**L-tripod cat**	6 (100%)	29 (100%)	1.00*
**R-tripod cat**	6 (100%)	29 (100%)	1.00*
**L-2 point cat:** Below normal	6 (100%)	26 (89.7%)	1.00*
Normal	0 (0%)	3 (10.3%)	-
**R-2 point Cat:** Below normal	6 (100%)	25 (86.2%)	1.00*
Normal	0 (0%)	4 (13.8%)	-

Missing values: at what year in = 0/23, presence of numbness or tingling while performing a procedure = 1/0.
*Fisher's exact test.
†T-test.
BMI, body mass index; CI, confidence interval; EGD, esophagogastroduodenoscopy; ERCP, endoscopic retrograde cholangiopancreatography; IQR interquartile range; QD, QuickDash; SD, standard deviation; Sx, symptom.

Appendix Table 6 Association between risk factors and any current pain.

Variable	Yes (N = 12)	No (N = 23)	*P* value
**Gender:** Female	4 (33.3%)	5 (21.7%)	0.69*
Male	8 (66.7%)	18 (78.3%)	-
**Age:** Mean (SD)	46.8 (11.7)	43.5 (10.5)	0.42†
Median (IQR)	42.5 (40.8-56.2)	42.0 (34.5-48.0)	-
Range	(30.0- 71.0)	(30.0- 64.0)	-
**Glove size:** Medium	3 (25%)	12 (52.2%)	0.26*
Small	3 (25%)	5 (21.7%)	-
Large/extra large	6 (50%)	6 (26.1%)	-
**Dominant hand:** Left	1 (8.3%)	0 (0%)	0.34*
Right	11 (91.7%)	23 (100%)	-
**Years performing procedures:** 0-5	2 (16.7%)	8 (34.8%)	0.25*
6-10	4 (33.3%)	7 (30.4%)	-
11-15	2 (16.7%)	0 (0%)	-
16-20	0 (0%)	3 (13%)	-
21-25	1 (8.3%)	3 (13%)	-
26-30	2 (16.7%)	1 (4.3%)	-
30-35	1 (8.3%)	1 (4.3%)	-
**BMI:** Mean (SD)	24.6 (2.7)	24.4 (3.2)	0.87†
Median (IQR)	24.0 (23.5-25.3)	24.4 (21.8-26.2)	-
Range	(21.0- 30.7)	(17.7- 29.5)	-
**time spent scoping:** < 10 hours	0 (0%)	4 (17.4%)	0.53*
10-20 hours	4 (33.3%)	8 (34.8%)	-
21-30 hours	6 (50%)	8 (34.8%)	-
30+ hours	2 (16.7%)	3 (13%)	-
**Number of procedures per week:** 0-20	1 (8.3%)	10 (43.5%)	0.007*
20-40	7 (58.3%)	12 (52.2%)	-
40-60	4 (33.3%)	0 (0%)	-
80+	0 (0%)	1 (4.3%)	-
**Type of procedure:** Colonoscopy	11 (91.7%)	20 (87%)	1.00*
EGD/EUS	0 (0%)	1 (4.3%)	-
ERCP	1 (8.3%)	2 (8.7%)	-
**Colonoscopy time:** 0%-40%	3 (25%)	6 (26.1%)	0.90*
40%-60%	5 (41.7%)	7 (30.4%)	-
60%-100%	4 (33.3%)	10 (43.5%)	-
**ERCP time:** 0%-20%	11 (91.7%)	21 (91.3%)	1.00*
20%-60%	1 (8.3%)	2 (8.7%)	-

Variable	Yes (N = 12)	No (N = 23)	*P* value
**EGD time:** 0%-20%	3 (25%)	3 (13%)	0.35*
20%-40%	6 (50%)	11 (47.8%)	-
40%-60%	3 (25%)	4 (17.4%)	-
60%-100%	0 (0%)	5 (21.7%)	-
**EUS time:** 0%-20%	11 (91.7%)	22 (95.7%)	1.00*
20%-40%	1 (8.3%)	1 (4.3%)	-
**Modifications - body position:** No	1 (8.3%)	2 (8.7%)	1.00*
Yes	11 (91.7%)	21 (91.3%)	-
**Activity level:** Moderate	8 (66.7%)	12 (52.2%)	0.69*
Mild/none	1 (8.3%)	4 (17.4%)	-
Extreme	3 (25%)	7 (30.4%)	-
**QD disability Sx score:** Mean (SD)	10.0 (7.7)	3.9 (9.0)	0.042†
Median (IQR)	10.2 (2.3-14.2)	2.3 (0.0- 2.3)	-
Range	(0.0- 25.0)	(0.0- 43.2)	-
**presence of numbness or tingling at night:** No	6 (50%)	23 (100%)	<0.001*
Yes	6 (50%)	0 (0%)	-
**Presence of numbness or tingling while performing a procedure:** No	9 (81.8%)	22 (95.7%)	0.24*
Yes	2 (18.2%)	1 (4.3%)	-
**Modified:** No	6 (50%)	23 (100%)	<0.001*
Yes	6 (50%)	0 (0%)	-
**Any positive provocative test:** No	2 (16.7%)	8 (34.8%)	0.43*
Yes	10 (83.3%)	15 (65.2%)	-
**Neck provocative test:** Negative	10 (83.3%)	21 (91.3%)	0.59*
Positive	2 (16.7%)	2 (8.7%)	-
**Shoulder provocative test:** Negative	5 (41.7%)	11 (47.8%)	0.73‡
Positive	7 (58.3%)	12 (52.2%)	-
**Back provocative test:** Negative	9 (75%)	14 (60.9%)	0.48*
Positive	3 (25%)	9 (39.1%)	-
**Elbow provocative test:** Negative	8 (66.7%)	21 (91.3%)	0.15*
Positive	4 (33.3%)	2 (8.7%)	-
**Wrist provocative test:** Negative	5 (41.7%)	17 (73.9%)	0.08*
Positive	7 (58.3%)	6 (26.1%)	-

Variable	Yes (N = 12)	No (N = 23)	*P* value
**Hand/thumb provocative test:** Negative	10 (83.3%)	22 (95.7%)	0.27*
Positive	2 (16.7%)	1 (4.3%)	-
**R-grip avg (lb):** Mean (SD)	82.6 (24.3)	82.2 (23.8)	0.96†
Median (IQR)	85.5 (64.2-102.7)	83.3 (69.7-93.5)	-
Range	(44.0- 126.0)	(38.3- 126.7)	-
**L-grip avg (lb):** Mean (SD)	79.7 (20.9)	75.9 (21.8)	0.62†
Median (IQR)	87.2 (64.3-95.0)	79.0 (61.5-92.0)	-
Range	(43.3- 104.7)	(32.0- 114.7)	-
**L-lat pinch:** Mean (SD)	19.1 (3.7)	18.3 (5.4)	0.62†
Median (IQR)	18.5 (17.0-20.5)	18.0 (15.5-21.5)	-
Range	(14.0- 27.0)	(9.0- 27.0)	-
**R-lat pinch:** Mean (SD)	20.0 (4.4)	18.8 (4.8)	0.48†
Median (IQR)	20.0 (16.5-22.5)	20.0 (16.0-21.0)	-
Range	(14.0- 28.0)	(9.0- 30.0)	-
**L-tripod:** Mean (SD)	14.2 (3.0)	13.7 (4.3)	0.71†
Median (IQR)	14.0 (12.8-16.0)	13.0 (10.5-17.0)	-
Range	(8.0- 20.0)	(5.0- 20.0)	-
**R-tripod:** Mean (SD)	15.8 (3.5)	14.1 (3.8)	0.22†
Median (IQR)	15.0 (13.8-18.2)	14.0 (11.0-16.0)	-
Range	(10.0- 22.0)	(8.0- 23.0)	-
**L-2 point:** Mean (SD)	10.2 (2.3)	10.2 (3.6)	0.96†
Median (IQR)	9.5 (9.0- 10.2)	10.0 (7.5-12.5)	-
Range	(8.0- 16.0)	(5.0- 18.0)	-
**R-2 point:** Mean (SD)	11.1 (3.5)	11.1 (3.0)	1.00†
Median (IQR)	10.0 (8.8-12.0)	11.0 (9.5-14.0)	-
Range	(8.0- 18.0)	(5.0- 17.0)	-
**R-grip cat:** Below normal	5 (41.7%)	3 (13%)	0.21*
Normal	4 (33.3%)	13 (56.5%)	-
Above Normal	3 (25%)	7 (30.4%)	-
**L-grip cat:** Below normal	5 (41.7%)	5 (21.7%)	0.07*
Normal	2 (16.7%)	13 (56.5%)	-
Above normal	5 (41.7%)	5 (21.7%)	-
**L-lat pinch cat:** Below Normal	8 (66.7%)	18 (78.3%)	0.69*
Normal	4 (33.3%)	5 (21.7%)	-

Variable	Yes (N = 12)	No (N = 23)	*P* value
**R-lat pinch cat:** Below Normal	8 (66.7%)	20 (87%)	0.20*
Normal	4 (33.3%)	3 (13%)	-
**L-tripod cat**	12 (100%)	23 (100%)	1.00*
**R-tripod cat**	12 (100%)	23 (100%)	1.00*
**L-2 point cat:** Below Normal	12 (100%)	20 (87%)	0.54*
Normal	0 (0%)	3 (13%)	-
**R-2 point cat:** Below Normal	10 (83.3%)	21 (91.3%)	0.59*
Normal	2 (16.7%)	2 (8.7%)	-

Missing values: at what year in = 0/23, presence of numbness or tingling while performing a procedure = 1/0.
*Fisher's exact test.
†T-test.
‡Chi-squared test.
BMI, body mass index; CI, confidence interval; EGD, esophagogastroduodenoscopy; ERCP, endoscopic retrograde cholangiopancreatography; IQR interquartile range; QD, QuickDash; SD, standard deviation; Sx, symptom.

Appendix Table 7 Association between risk factors and modified endoscopy technique due to your pain/injury.

Variable	Yes (N = 6)	No (N = 29)	*P* value
**gender:** Female	2 (33.3%)	7 (24.1%)	0.64*
Male	4 (66.7%)	22 (75.9%)	-
**age:** Mean (SD)	41.2 (9.2)	45.3 (11.2)	0.36†
Median (IQR)	41.0 (36.2-42.8)	43.0 (36.0-51.0)	-
Range	(30.0- 57.0)	(30.0- 71.0)	-
**Glove size:** Medium	2 (33.3%)	13 (44.8%)	0.73*
Small	1 (16.7%)	7 (24.1%)	-
Large/extra large	3 (50%)	9 (31%)	-
**Dominant hand:** Left	1 (16.7%)	0 (0%)	0.17*
Right	5 (83.3%)	29 (100%)	-
**Years performing procedures:** 0-5	2 (33.3%)	8 (27.6%)	1.00*
6-10	3 (50%)	8 (27.6%)	-
11-15	0 (0%)	2 (6.9%)	-
16-20	0 (0%)	3 (10.3%)	-
21-25	1 (16.7%)	3 (10.3%)	-
26-30	0 (0%)	3 (10.3%)	-
30-35	0 (0%)	2 (6.9%)	-
**BMI:** Mean (SD)	24.4 (3.5)	24.5 (3.0)	0.95†
Median (IQR)	23.7 (22.0-25.3)	24.4 (22.4-25.8)	-
Range	(21.0- 30.7)	(17.7- 29.5)	-
**time spent scoping:** < 10 hours	0 (0%)	4 (13.8%)	0.50*
10-20 hours	2 (33.3%)	10 (34.5%)	-
21-30 hours	2 (33.3%)	12 (41.4%)	-
30+ hours	2 (33.3%)	3 (10.3%)	-
**Number of procedures per week:** 0-20	0 (0%)	11 (37.9%)	0.009*
20-40	3 (50%)	16 (55.2%)	-
40-60	3 (50%)	1 (3.4%)	-
80+	0 (0%)	1 (3.4%)	-
**Type of procedure:** Colonoscopy	6 (100%)	25 (86.2%)	1.00*
EGD/EUS	0 (0%)	1 (3.4%)	-
ERCP	0 (0%)	3 (10.3%)	-
**Colonoscopy time:** 0%-40%	1 (16.7%)	8 (27.6%)	0.73*
40%-60%	3 (50%)	9 (31%)	-
60%-100%	2 (33.3%)	12 (41.4%)	-
**ERCP time:** 0%-20%	6 (100%)	26 (89.7%)	1.00*

Variable	Yes (N = 6)	No (N = 29)	*P* value
20%-60%	0 (0%)	3 (10.3%)	-
**EGD Time:** 0%-20%	2 (33.3%)	4 (13.8%)	0.36*
20%-40%	2 (33.3%)	15 (51.7%)	-
40%-60%	2 (33.3%)	5 (17.2%)	-
60%-100%	0 (0%)	5 (17.2%)	-
**EUS time:** 0%-20%	6 (100%)	27 (93.1%)	1.00*
20%-40%	0 (0%)	2 (6.9%)	-
**Modifications - Body position:** No	0 (0%)	3 (10.3%)	1.00*
Yes	6 (100%)	26 (89.7%)	-
**Activity level:** Moderate	3 (50%)	17 (58.6%)	1.00*
Mild/none	1 (16.7%)	4 (13.8%)	-
Extreme	2 (33.3%)	8 (27.6%)	-
**At what year in:** 0-5	5 (83.3%)	0 (0%)	0.004*
6-10	0 (0%)	2 (33.3%)	-
11-15	0 (0%)	3 (50%)	-
16-20	1 (16.7%)	0 (0%)	-
26-30	0 (0%)	1 (16.7%)	-
**QD disability Sx score:** Mean (SD)	7.6 (5.9)	5.6 (9.5)	0.53†
Median (IQR)	7.9 (3.4-10.8)	2.3 (0.0-4.5)	-
Range	(0.0- 15.9)	(0.0-43.2)	-
**Presence of numbness or tingling at night:** No	2 (33.3%)	27 (93.1%)	0.004*
Yes	4 (66.7%)	2 (6.9%)	-
**Presence of numbness or tingling while performing a procedure:** No	4 (80%)	27 (93.1%)	0.39*
Yes	1 (20%)	2 (6.9%)	-
**Any current pain:** No	0 (0%)	23 (79.3%)	< 0.001*
Yes	6 (100%)	6 (20.7%)	-
**Any positive provocative test:** No	1 (16.7%)	9 (31%)	0.65*
Yes	5 (83.3%)	20 (69%)	-
**Neck provocative test:** Negative	5 (83.3%)	26 (89.7%)	0.55*
Positive	1 (16.7%)	3 (10.3%)	-
**Shoulder provocative test:** Negative	2 (33.3%)	14 (48.3%)	0.67*
Positive	4 (66.7%)	15 (51.7%)	-
**Back provocative test:** Negative	4 (66.7%)	19 (65.5%)	1.00*
Positive	2 (33.3%)	10 (34.5%)	-

Variable	Yes (N = 6)	No (N = 29)	*P* value
**Elbow provocative test:** Negative	4 (66.7%)	25 (86.2%)	0.27*
Positive	2 (33.3%)	4 (13.8%)	-
**Wrist provocative test:** Negative	3 (50%)	19 (65.5%)	0.65*
Positive	3 (50%)	10 (34.5%)	-
**Hand/thumb provocative test:** Negative	5 (83.3%)	27 (93.1%)	0.44*
Positive	1 (16.7%)	2 (6.9%)	-
**R-grip avg (lb):** Mean (SD)	75.4 (18.8)	83.8 (24.5)	0.37†
Median (IQR)	75.2 (62.8-85.6)	86.3 (71.0-103.3)	-
Range	(52.0- 102.3)	(38.3- 126.7)	-
**L-grip avg (lb):** Mean (SD)	78.3 (21.8)	77.0 (21.6)	0.89†
Median (IQR)	83.7 (59.6-92.2)	81.7 (63.0-95.7)	-
Range	(51.3- 104.7)	(32.0- 114.7)	-
**L-lat pinch:** Mean (SD)	19.7 (4.5)	18.3 (4.9)	0.54†
Median (IQR)	19.0 (17.2-21.5)	18.0 (16.0-21.0)	-
Range	(14.0- 27.0)	(9.0- 27.0)	-
**R-lat pinch:** Mean (SD)	20.5 (5.0)	19.0 (4.6)	0.51†
Median (IQR)	20.0 (17.8-23.0)	20.0 (16.0-22.0)	-
Range	(14.0- 28.0)	(9.0- 30.0)	-
**L-tripod:** Mean (SD)	14.5 (4.2)	13.7 (3.8)	0.69†
Median (IQR)	15.0 (12.5-16.8)	14.0 (12.0-16.0)	-
Range	(8.0- 20.0)	(5.0- 20.0)	-
**R-tripod:** Mean (SD)	16.8 (3.8)	14.2 (3.6)	0.17†
Median (IQR)	16.5 (14.2-19.5)	14.0 (12.0-16.0)	-
Range	(12.0- 22.0)	(8.0- 23.0)	-
**L-2 point:** Mean (SD)	10.7 (3.2)	10.1 (3.2)	0.71†
Median (IQR)	9.5 (8.2-12.2)	10.0 (9.0-11.0)	-
Range	(8.0- 16.0)	(5.0- 18.0)	-
**R-2 point:** Mean (SD)	11.5 (3.6)	11.0 (3.1)	0.76†
Median (IQR)	11.0 (9.2-12.0)	10.0 (9.0-14.0)	-
Range	(8.0- 18.0)	(5.0- 18.0)	-
**R-grip cat:** Below normal	1 (16.7%)	7 (24.1%)	1.00*
Normal	3 (50%)	14 (48.3%)	-
Above normal	2 (33.3%)	8 (27.6%)	-
**L-grip cat:** Below bormal	1 (16.7%)	9 (31%)	0.13*

Variable	Yes (N = 6)	No (N = 29)	*P* value
Normal	1 (16.7%)	14 (48.3%)	-
Above normal	4 (66.7%)	6 (20.7%)	-
**L-lat pinch cat:** Below normal	4 (66.7%)	22 (75.9%)	0.64*
Normal	2 (33.3%)	7 (24.1%)	-
**R-lat pinch cat:** Below normal	4 (66.7%)	24 (82.8%)	0.58*
Normal	2 (33.3%)	5 (17.2%)	-
**L-tripod cat**	6 (100%)	29 (100%)	1.00*
**R-tripod cat**	6 (100%)	29 (100%)	1.00*
**L-2 point cat:** Below normal	6 (100%)	26 (89.7%)	1.00*
Normal	0 (0%)	3 (10.3%)	-
**R-2 point cat:** Below normal	5 (83.3%)	26 (89.7%)	0.55*
Normal	1 (16.7%)	3 (10.3%)	-

Missing values: at what year in = 0/23, presence of numbness or tingling while performing a procedure = 1/0.

*Fisher's exact test.

†T-test.

BMI, body mass index; CI, confidence interval; EGD, esophagogastroduodenoscopy; ERCP, endoscopic retrograde cholangiopancreatography; IQR interquartile range; QD, QuickDash; SD, standard deviation; Sx, symptom.

Appendix Table 8 Association between risk factors and any positive provocative test.

Variable	Yes (N = 25)	No (N = 10)	*P* value
**Gender:** Female	5 (20%)	4 (40%)	0.39[*]
Male	20 (80%)	6 (60%)	-
**Age:** Mean (SD)	45.8 (11.5)	41.6 (8.9)	0.26[†]
Median (IQR)	43.0 (36.0-56.0)	41.5 (36.2-43.8)	-
Range	(30.0-71.0)	(30.0-63.0)	-
**Glove size:** Medium	10 (40%)	5 (50%)	0.12[*]
Small	4 (16%)	4 (40%)	-
Large/extra large	11 (44%)	1 (10%)	-
**Dominant hand:** Left	1 (4%)	0 (0%)	1.00[*]
Right	24 (96%)	10 (100%)	-
**Years performing procedures:** 0-5	6 (24%)	4 (40%)	0.64[*]
6-10	7 (28%)	4 (40%)	-
11-15	2 (8%)	0 (0%)	-
16-20	2 (8%)	1 (10%)	-
21-25	4 (16%)	0 (0%)	-
26-30	3 (12%)	0 (0%)	-
30-35	1 (4%)	1 (10%)	-
**BMI:** Mean (SD)	25.0 (2.7)	23.2 (3.4)	0.15[†]
Median (IQR)	24.4 (23.7-25.8)	22.6 (21.1-25.1)	-
Range	(20.1-30.7)	(17.7-28.3)	-
**time spent scoping:** < 10 hours	4 (16%)	0 (0%)	0.69[*]
10-20 hours	8 (32%)	4 (40%)	-
21-30 hours	10 (40%)	4 (40%)	-
30+ hours	3 (12%)	2 (20%)	-
**Number of procedures per week:** 0-20	9 (36%)	2 (20%)	0.43[*]
20-40	13 (52%)	6 (60%)	-
40-60	3 (12%)	1 (10%)	-
80+	0 (0%)	1 (10%)	-
**Type of procedure:** Colonoscopy	23 (92%)	8 (80%)	0.43[*]
EGD/EUS	1 (4%)	0 (0%)	-
ERCP	1 (4%)	2 (20%)	-
**Colonoscopy time:** 0 - 40%	7 (28%)	2 (20%)	0.80[*]
40%-60%	9 (36%)	3 (30%)	-
60%-100%	9 (36%)	5 (50%)	-
**ERCP time:** 0%-20%	24 (96%)	8 (80%)	0.19[*]
20%-60%	1 (4%)	2 (20%)	-

Variable	Yes (N = 25)	No (N = 10)	*P* value
**EGD time:** 0%-20%	4 (16%)	2 (20%)	0.86*
20%-40%	13 (52%)	4 (40%)	-
40%-60%	5 (20%)	2 (20%)	-
60%-100%	3 (12%)	2 (20%)	-
**EUS time:** 0%-20%	24 (96%)	9 (90%)	0.50*
20%-40%	1 (4%)	1 (10%)	-
**Modifications - Body position:** No	3 (12%)	0 (0%)	0.54*
Yes	22 (88%)	10 (100%)	-
**Activity level:** Moderate	16 (64%)	4 (40%)	0.23*
Mild/None	4 (16%)	1 (10%)	-
Extreme	5 (20%)	5 (50%)	-
**At what year in:** 0-5	4 (40%)	1 (50%)	0.77*
6-10	1 (10%)	1 (50%)	-
11-15	3 (30%)	0 (0%)	-
16-20	1 (10%)	0 (0%)	-
26-30	1 (10%)	0 (0%)	-
**QD disability Sx score:** Mean (SD)	7.2 (10.0)	2.9 (4.4)	0.09†
Median (IQR)	2.3 (2.3- 9.1)	1.1 (0.0- 4.0)	-
Range	(0.0- 43.2)	(0.0- 13.6)	-
**Presence of numbness or tingling at night:** No	20 (80%)	9 (90%)	0.65*
Yes	5 (20%)	1 (10%)	-
**Presence of numbness or tingling while performing a procedure:** No	21 (87.5%)	10 (100%)	0.54*
Yes	3 (12.5%)	0 (0%)	-
**Any current pain:** No	15 (60%)	8 (80%)	0.43*
Yes	10 (40%)	2 (20%)	-
**Modified:** No	20 (80%)	9 (90%)	0.65*
Yes	5 (20%)	1 (10%)	-
**Neck provocative test:** Negative	21 (84%)	10 (100%)	0.30*
Positive	4 (16%)	0 (0%)	-
**Shoulder provocative test:** Negative	6 (24%)	10 (100%)	< 0.001*
Positive	19 (76%)	0 (0%)	-
**Back provocative test:** Negative	13 (52%)	10 (100%)	0.007*
Positive	12 (48%)	0 (0%)	-
**Elbow provocative test:** Negative	19 (76%)	10 (100%)	0.15*
Positive	6 (24%)	0 (0%)	-

Variable	Yes (N = 25)	No (N = 10)	*P* value
**Wrist provocative test:** Negative	12 (48%)	10 (100%)	0.005*
Positive	13 (52%)	0 (0%)	-
**Hand/thumb provocative test:** Negative	22 (88%)	10 (100%)	0.54*
Positive	3 (12%)	0 (0%)	-
**R-grip Avg (lb):** Mean (SD)	84.8 (22.8)	76.2 (25.7)	0.37†
Median (IQR)	85.7 (71.0-102.3)	81.5 (60.0-91.8)	-
Range	(44.0- 126.7)	(38.3-114.7)	-
**L-grip Avg (lb):** Mean (SD)	80.9 (19.5)	68.0 (23.9)	0.15†
Median (IQR)	83.0 (68.0-96.7)	74.0 (51.5-87.5)	-
Range	(43.3- 114.7)	(32.0- 98.3)	-
**L-lat pinch:** Mean (SD)	18.9 (4.2)	17.8 (6.4)	0.63†
Median (IQR)	18.0 (16.0-21.0)	19.5 (11.8-22.5)	-
Range	(11.0- 27.0)	(9.0- 25.0)	-
**R-lat pinch:** Mean (SD)	19.8 (4.1)	17.8 (5.8)	0.34†
Median (IQR)	20.0 (16.0-22.0)	19.0 (13.2-22.2)	-
Range	(14.0- 30.0)	(9.0- 25.0)	-
**L-tripod:** Mean (SD)	14.0 (3.3)	13.4 (5.1)	0.72†
Median (IQR)	14.0 (12.0-16.0)	13.5 (10.5-16.8)	-
Range	(8.0- 20.0)	(5.0- 20.0)	-
**R-tripod:** Mean (SD)	15.1 (3.6)	13.7 (4.0)	0.36†
Median (IQR)	15.0 (13.0-17.0)	14.5 (10.8-15.8)	-
Range	(10.0- 23.0)	(8.0- 19.0)	-
**L-2 point:** Mean (SD)	10.2 (2.9)	10.2 (3.9)	1.00†
Median (IQR)	10.0 (8.0-11.0)	9.5 (9.0-11.8)	-
Range	(6.0- 17.0)	(5.0- 18.0)	-
**R-2 point:** Mean (SD)	10.8 (2.6)	11.7 (4.2)	0.56†
Median (IQR)	10.0 (9.0-12.0)	11.5 (10.0-14.0)	-
Range	(8.0- 18.0)	(5.0- 18.0)	-
**R-grip cat:** Below normal	7 (28%)	1 (10%)	0.55*
Normal	11 (44%)	6 (60%)	-
Above normal	7 (28%)	3 (30%)	-
**L-grip cat:** Below normal	9 (36%)	1 (10%)	0.11*
Normal	8 (32%)	7 (70%)	-
Above normal	8 (32%)	2 (20%)	-

Variable	Yes (N = 25)	No (N = 10)	*P* value
**L-lat pinch cat:** Below Normal	18 (72%)	8 (80%)	1.00*
Normal	7 (28%)	2 (20%)	-
**R-lat pinch cat:** Below Normal	20 (80%)	8 (80%)	1.00*
Normal	5 (20%)	2 (20%)	-
**L-tripod cat**	25 (100%)	10 (100%)	1.00*
**R-tripod cat**	25 (100%)	10 (100%)	1.00*
**L-2 point cat:** Below Normal	24 (96%)	8 (80%)	0.19*
Normal	1 (4%)	2 (20%)	-
**R-2 point cat:** Below Normal	24 (96%)	7 (70%)	0.06*
Normal	1 (4%)	3 (30%)	-

Missing values: at what year in=15/8, presence of numbness or tingling while performing a procedure=1/0.
*Fisher's exact test,
†T-test.
BMI, body mass index; CI, confidence interval; EGD, esophagogastroduodenoscopy; ERCP, endoscopic retrograde cholangiopancreatography; IQR interquartile range; QD, QuickDash; SD, standard deviation; Sx, symptom.

Appendix Table 9 Linear regression after Lasso variable selection using for QD disability symptom score.

	Estimate (95% CI)	*P* value
Age	0.06 (-0.78-0.91)	0.88
Glove size small	1.77 (-12.82-16.36)	0.8
Glove size large/extra large	-2.37 (-11.76-7.02)	0.59
Years performing procedures 0-5	-10.74 (-24.81-3.32)	0.12
Years performing procedures 11-15	0.79 (-20.48-22.05)	0.94
Years performing procedures 16-20	14.12 (-1.85-30.1)	0.08
Years performing procedures 21-25	6.54 (-11.62-24.71)	0.45
Years performing procedures 26-30	11.04 (-11.77-33.85)	0.31
Years performing procedures 30-35	-3.31 (-33.56-26.95)	0.82
Time spent scoping < 10 hours	-2.84 (-19.55-13.87)	0.72
Time spent scoping 21-30 hours	5.29 (-6.42-17)	0.34
Time spent scoping 30+ hours	-1.58 (-15.28-12.11)	0.81
Number of procedures per week 0-20	5.79 (-7.58-19.16)	0.36
Number of procedures per week 40-60	15.99 (-1.32-33.3)	0.07
Number of procedures each week 80+	8.86 (-15.68-33.4)	0.45
Colonoscopy time 40%-60%	-5.96 (-18.83-6.91)	0.33
Colonoscopy time 60%-100%	-10.09 (-26.59-6.41)	0.21
EGD time 20%-40%	-8.6 (-23.94-6.73)	0.24
EGD time 40%-60%	5.01 (-17.9-27.93)	0.64
EGD Time 60%-100%	2.76 (-20.69-26.21)	0.8
	5.24 (-9.15-19.62)	0.44
Activity level mild/none		
Activity level extreme	-2.77 (-13.63-8.1)	0.59

Reference: Gender = Female, colonoscopy time = 0%-40%, ERCP time = 0%-20%, EGD time = 0%-20%, EUS time = 0%-20%, glove size = medium, activity level = moderate, years performing procedures = 0-6, time spent scoping = 10-20, number of procedures each week

= 20-40.
CI, confidence interval; EGD, esophagogastroduodenoscopy; ERCP, endoscopic retrograde cholangiopancreatography.

Appendix Table 10 Linear regression after Lasso variable selection for R-grip avg (lb).

	Estimate (95% CI)	*P* value
Gender male	18.34 (-13.37-50.04)	0.24
Glove size small	-19.04 (-52.68-14.59)	0.25
Glove size large/extra large	-6.89 (-25.23-11.46)	0.44
Years performing procedures 0-5	-5.58 (-25.01-13.85)	0.55
Years performing procedures 11-15	-2.8 (-37.79-32.18)	0.87
Years performing procedures 16-20	-18.06 (-49.02-12.91)	0.24
Years performing procedures 21-25	-9.99 (-36.26-16.28)	0.44
Years performing procedures 26-30	24.5 (-5.36-54.36)	0.1
Years performing procedures 30-35	9.2 (-27.76-46.16)	0.61
Time spent scoping < 10 hours	-5.17 (-35.14-24.81)	0.72
Time spent scoping 21-30 hours	2.42 (-16.95-21.79)	0.8
Time spent scoping 30+ hours	7.33 (-22.98-37.64)	0.62
Number of procedures per week 0-20	-12.04 (-35.55-11.47)	0.3
Number of procedures per week 40-60	2.71 (-29.06-34.48)	0.86
Number of procedures per week 80+	-6.22 (-53.53-41.09)	0.79

Reference: Gender = female, colonoscopy time = 0%-40%, ERCP time = 0%-20%, EGD time = 0%-20%, EUS time = 0%-20%, glove size = medium, activity level = moderate.
CI, confidence interval; EGD, esophagogastroduodenoscopy; ERCP, endoscopic retrograde cholangiopancreatography.

Appendix Table 11 Linear regression after Lasso variable selection for L-grip avg (lb).

	Estimate (95% CI)	*P* value
Gender male	10.69 (-13.58-34.95)	0.37
Glove size small	-23.32 (-50.72-4.08)	0.09
Glove size large/extra large	-6.73 (-21.75-8.3)	0.37
Years performing procedures 0-5	-4.79 (-21.13-11.55)	0.55
Years performing procedures 11-15	0.7 (-30.5-31.91)	0.96
Years performing procedures 16-20	-18.83 (-43.52-5.86)	0.13
Years performing procedures 21-25	-1.71 (-23.89-20.48)	0.88
Years performing procedures 26-30	20.41 (-3.15-43.96)	0.09
Years performing procedures 30-35	0.06 (-28.19-28.32)	1

Reference: gender = female, colonoscopy time = 0%-40%, ERCP time = 0%-20%, EGD time = 0%-20%, EUS time = 0%-20%, glove size = medium, activity level = moderate.
CI, confidence interval; EGD, esophagogastroduodenoscopy; ERCP, endoscopic retrograde cholangiopancreatography.

Appendix Table 12 Linear regression after Lasso variable selection using for L-Lat pinch.

	**Estimate (95% CI)**	***P* value**
Gender male	7.65 (4.77-10.53)	< 0.001
Age	-0.11 (-0.23-0.01)	0.06
Time spent scoping < 10 hours	-1.53 (-6.02-2.96)	0.49
Time spent scoping 21-30 hours	-0.4 (-3.34-2.54)	0.78
Time spent scoping 30+ hours	1.86 (-2.97-6.68)	0.44
Number of procedures per week 0-20	-2.49 (-5.75-0.77)	0.13
Number of procedures per week 40-60	0.53 (-3.53-4.58)	0.79
Number of procedures per week 80+	0.33 (-7.29-7.94)	0.93
ERCP time 20%-60%	-3.33 (-7.43-0.76)	0.11

Reference: gender = female, colonoscopy time = 0%-40%, ERCP time = 0%-20%, EGD time = 0%-20%, EUS time = 0%-20%, glove size = medium, activity level = moderate.

CI, confidence interval; EGD, esophagogastroduodenoscopy; ERCP, endoscopic retrograde cholangiopancreatography.

Appendix Table 13 Linear regression after Lasso variable selection using for R-lat pinch.

	**Estimate (95% CI)**	***P* value**
Gender male	7.61 (4.87-10.35)	< 0.001
Age	-0.08 (-0.19-0.04)	0.18
Time spent scoping < 10 hours	-1.68 (-5.96-2.6)	0.43
Time spent scoping 21-30 hours	-0.72 (-3.52-2.08)	0.6
Time spent scoping 30+ hours	0.84 (-3.75-5.43)	0.71
Number of procedures per week 0-20	-4.49 (-7.59 to -1.38)	0.01
Number of procedures per week 40-60	1.08 (-2.78-4.93)	0.57
Number of procedures per week 80+	-0.79 (-8.04-6.45)	0.82
ERCP time 20%-60%	-3.38 (-7.28-0.52)	0.09

Reference: gender = female, colonoscopy time = 0%- 40%, ERCP time = 0%-20%, EGD time = 0%-20%, EUS time = 0%-20%, glove size = medium, activity level = moderate.

CI, confidence interval; EGD, esophagogastroduodenoscopy; ERCP, endoscopic retrograde cholangiopancreatography.

Appendix Table 14 Linear regression after Lasso variable selection using for L-tripod.

	Estimate (95% CI)	*P* value
Gender male	4.76 (1.08-8.44)	0.01
Age	-0.16 (-0.27 to -0.05)	0.01
Glove size small	2.28 (-1.54-6.1)	0.23
Glove size large/extra large	2.05 (-0.33-4.42)	0.09
Time spent scoping < 10 hours	-3.74 (-7.58-0.1)	0.06
Time spent scoping 21-30 hours	3.91 (1.43-6.39)	0
Time spent scoping 30+ hours	3.31 (-0.61-7.24)	0.09
Number of procedures per week 0-20	1.72 (-1.19-4.63)	0.23
Number of procedures per week 40-60	-1.52 (-5.23-2.18)	0.4
Number of procedures each week 80+	-5.84 (-12.19-0.51)	0.07
Activity level mild/none	-3.49 (-6.75 to -0.22)	0.04
Activity level extreme	1.78 (-0.66-4.23)	0.14

Reference: gender = female, colonoscopy time = 0%-40%, ERCP time = 0%-20%, EGD time = 0%-20%, EUS time = 0%-20%, glove size = medium, activity level = moderate.
CI, confidence interval; EGD, esophagogastroduodenoscopy; ERCP, endoscopic retrograde cholangiopancreatography.

Appendix Table 15 Linear regression after Lasso variable selection using for R-tripod.

	**Estimate (95% CI)**	***P* value**
Gender male	3.5 (0.62-6.37)	0.02
Age	-0.13 (-0.21 to -0.05)	0
Glove size small	-0.22 (-3.08-2.65)	0.88
Glove size large/extra large	2.83 (0.94-4.72)	0.01
Time spent scoping < 10 hours	-5.52 (-8.41 to -2.62)	< 0.001
Time spent scoping 21-30 hours	0.7 (-1.23-2.63)	0.46
Time spent scoping 30+ hours	2.05 (-1.09-5.18)	0.19
Number of procedures per week 0-20	-1.78 (-3.91-0.35)	0.1
Number of procedures per week 40-60	-1.42 (-4.4-1.56)	0.33
Number of procedures per week 80+	-6.35 (-11.29 to -1.41)	0.01
ERCP time 20%-60%	-3.27 (-5.92 to -0.62)	0.02

Reference: gender = female, colonoscopy time = 0%-40%, ERCP time = 0%-20%, EGD time = 0%-20%, EUS time = 0%-20%, glove size = medium, activity level = moderate.

CI, confidence interval; EGD, esophagogastroduodenoscopy; ERCP, endoscopic retrograde cholangiopancreatography.

Appendix Table 16 Linear regression after Lasso variable selection using for L-2 point.

	Estimate (95% CI)	*P* value
Gender male	2.47 (-1.69-6.64)	0.22
Age	-0.27 (-0.46 to -0.07)	0.01
Glove size small	-2.46 (-7.1-2.18)	0.27
Glove size large/extra large	2.67 (0.23-5.12)	0.03
Years performing procedures 0-5	-0.53 (-4.03-2.97)	0.75
Years performing procedures 11-15	1.75 (-3.75-7.24)	0.5
Years performing procedures 16-20	-2.17 (-6.65-2.3)	0.31
Years performing procedures 21-25	2.06 (-2.76-6.87)	0.37
Years performing procedures 26-30	-0.76 (-6.41-4.89)	0.78
Years performing procedures 30-35	5.82 (-1.34-12.98)	0.1
Time spent scoping < 10 hours	-2.52 (-6.76-1.71)	0.22
Time spent scoping 21-30 hours	2.03 (-0.72-4.78)	0.13
Time spent scoping 30+ hours	1.5 (-2.32-5.31)	0.41
Number of procedures per week 0-20	0.04 (-3.37-3.46)	0.98
Number of procedures per week 40-60	-4.02 (-9.1-1.06)	0.11
Number of procedures per week 80+	-5.34 (-11.6-0.92)	0.09
ERCP time 20%-60%	-1.75 (-5.55-2.05)	0.33
EGD time 20%-40%	-1.05 (-4.71-2.61)	0.54
EGD time 40%-60%	-2.33 (-6.93-2.27)	0.29
EGD time 60%-100%	-1.14 (-5.73-3.45)	0.6
Activity level mild/none	-1.12 (-4.68-2.45)	0.51
Activity level extreme	1.87 (-0.76-4.51)	0.15

Reference: gender = female, colonoscopy time = 0%-40%, ERCP time = 0%-20%, EGD time = 0%-20%, EUS time = 0%-20%, glove size = medium, activity level = moderate.
CI, confidence interval; EGD, esophagogastroduodenoscopy; ERCP, endoscopic retrograde cholangiopancreatography.

Appendix Table 17 Linear regression after Lasso variable selection using for R-2point.

	**Estimate (95% CI)**	***P* value**
Gender male	4.42 (1.89-6.96)	0
Age	-0.21 (-0.4 to -0.02)	0.03
Years performing procedures 0-5	-0.99 (-3.52-1.55)	0.42
Years performing procedures 11-15	0.47 (-3.75-4.7)	0.82
Years performing procedures 16-20	1.5 (-2.41-5.4)	0.43
Years performing procedures 21-25	0.92 (-2.8-4.63)	0.61
Years performing procedures 26-30	1.64 (-2.91-6.2)	0.46
Years performing procedures 30-35	5.99 (-0.39-12.38)	0.06
BMI	0.46 (0.06-0.86)	0.03
Time spent scoping < 10 hours	-5.52 (-9.68 to -1.36)	0.01
Time spent scoping 21-30 hours	-0.2 (-3.04-2.63)	0.88
Time spent scoping 30+ hours	-0.11 (-3.47-3.26)	0.95
ERCP time 20%-60%	-2 (-5.26-1.25)	0.21
Modifications - body position yes	2.73 (-1.74-7.2)	0.22
Activity level mild/none	0.31 (-2.62-3.24)	0.83
Activity level extreme	1.98 (-0.29-4.26)	0.08

Reference: gender = female, colonoscopy time = 0%-40%, ERCP time = 0%-20%, EGD time = 0%-20%, EUS time = 0%-20%, glove size = medium, activity level = moderate.

BMI, body mass index; CI, confidence interval; EGD, esophagogastroduodenoscopy; ERCP, endoscopic retrograde cholangiopancreatography.
